# Pareto-Based Diagnostics and Selection for Mechanics–Synergy Trade-Offs in Unmeasured Muscle Activation Reconstruction

**DOI:** 10.3390/bioengineering13030293

**Published:** 2026-03-01

**Authors:** Po-Hsien Jiang, Kuei-Yuan Chan

**Affiliations:** Department of Mechanical Engineering, National Taiwan University, Taipei 10617, Taiwan

**Keywords:** musculoskeletal simulation, muscle activation reconstruction, muscle synergy, non-negative matrix factorization, multi-objective optimization, inverse problems

## Abstract

Background: Reconstructing full muscle activation trajectories from sparse measurements is underdetermined: many activation patterns can explain similar joint moments, and purely mechanical inverse formulations can yield non-physiological solutions. Methods: We propose a synergy-informed, physics-constrained framework to reconstruct unmeasured muscle activations when only a subset of muscles is observed. A synergy reconstruction prior (SynRc) is obtained by identifying a synergy basis from proxy activations via non-negative matrix factorization (NMF) and estimating time-varying synergy excitations from measured channels. Unmeasured activations are then solved via bound-constrained multi-objective optimization that jointly minimizes (i) normalized joint-moment error between OpenSim forward-computed moments and inverse-dynamics moments and (ii) deviation from the SynRc prior, with an optional smoothness refinement stage. Results: Verification on synthetic OpenSim Arm26 (2-DOF) cases with known ground truth shows that $J_{1}$-dominant selections from the stage-I Pareto set reduce normalized joint-moment error from 0.154 (SynRc-only) to ≈0.138, at the cost of larger deviation from the synergy prior. These Pareto diagnostics expose identifiability and selection sensitivity under sparse measurements when ground truth is unavailable. Conclusions: The proposed framework makes mechanics–synergy trade-offs explicit and provides structured diagnostics and selection guidance for sparse-measurement scenarios.

## 1. Introduction

Estimating muscle activation trajectories remains difficult when experimental sensing is sparse (e.g., sparse EMG). Reconstructing a full set of muscle activations from a small number of measured channels is fundamentally underdetermined: different activation patterns can yield similar net joint moments due to muscular redundancy and the nonlinear mapping from activation to musculotendon force [1,2]. As a result, purely mechanics-based inverse formulations can admit many feasible solutions, and additional structure (physiological priors, measurement constraints, or regularization) is required to obtain interpretable estimates.

Mechanics-based approaches such as static optimization (SO) and musculoskeletal optimal control enforce joint-moment consistency through a forward model [3,4,5,6]. These methods work well when model fidelity is high, but can be sensitive to modeling and measurement errors, and when only a subset of muscles is instrumented, additional assumptions are still necessary to infer unmeasured activations. EMG-driven and hybrid formulations incorporate measured excitations to improve physiological plausibility [7,8,9], but they still require design choices about how strongly to trust EMG versus physics when measurements are sparse or mismatched.

Synergy-based extrapolation leverages low-dimensional structure to infer unmeasured channels and can be effective when synergy structure is transferable [10,11,12,13,14,15,16,17,18]. However, synergy reconstruction alone does not guarantee joint-moment consistency in a musculoskeletal forward model, and fixed scalarization weights can obscure how reconstructed activations vary with modeling assumptions.

We propose a synergy-informed, physics-constrained approach that treats joint-moment consistency and neural consistency as competing objectives. First, a synergy reconstruction prior (SynRc) is computed by identifying a synergy basis from proxy activations via non-negative matrix factorization (NMF) and estimating time-varying synergy excitations from measured channels via a bounded quadratic program with temporal regularization. Second, unmeasured activations are estimated by solving a bound-constrained multi-objective inverse problem that trades off (i) normalized joint-moment error $J_{1}$ and (ii) deviation from the SynRc prior $J_{2}$, followed by an optional local refinement stage with a smoothness penalty. The resulting stage-I Pareto set makes the mechanics–synergy trade-off explicit and enables diagnostics and task-dependent solution selection when ground truth is unavailable.

*Scope and claims:* This paper does **not** claim universally accurate activation reconstruction across subjects and tasks, nor does it provide experimental validation or real-time deployment. Instead, it contributes a diagnostic and selection framework that exposes mechanics–synergy trade-offs via Pareto geometry and provides practical selection guidance under sparse measurements.

*Intended use:* The following framework targets offline analyses where interpretability and joint-moment fidelity matter under sparse observations (e.g., retrospective simulation studies or research workflows with sparse EMG) and where users need to assess identifiability and selection sensitivity rather than obtain a single black-box estimate.

The main contributions are as follows:

- A two-stage reconstruction framework combining a synergy reconstruction prior (SynRc) and a mechanics-constrained multi-objective inverse problem with optional smoothness refinement;
- A Pareto-based diagnostic and selection workflow (utopia-closest + neighborhood sensitivity) and a practical reporting template for applications without ground truth;
- Synthetic verification and stress tests illustrating how trade-off geometry changes across observability regimes (i.e., how sensitive joint moments are to unmeasured activations) and under noise/mismatch;
- A minimal reduced-dimension parameterization demo that motivates scalable implementations.

We emphasize that the present study is a verification on synthetic data with known ground truth [19]. The synthetic setting enables controlled analysis of identifiability and trade-offs but can underestimate the impact of experimental noise, modeling mismatch, and subject-specific variability; these limitations are discussed in Section 4. The remainder of this paper is organized as follows: Section 2 presents the proposed formulation and solver, Section 3 reports results and diagnostics, Section 4 discusses implications and limitations, and Section 5 concludes.

## 2. Methods

### 2.1. Nomenclature

- A(t) muscle activation vector at time *t* (bounded in ${\left[0,1\right]}^{M}$).
- $A^{\mathrm{meas}}\left(t\right),A^{\mathrm{miss}}\left(t\right)$ measured and unmeasured activations.
- $E_{\mathrm{meas}}$ measured-channel SynRc reconstruction error.
- h(t) synergy excitation at time *t* (bounded in ${\left[0,1\right]}^{r}$).
- $J_{1},J_{2},J_{3}$ joint-moment-consistency, neural-consistency, and smoothness objectives.
- **Observability regime**: The degree to which joint moments are informative about unmeasured activations under fixed kinematics. High observability means joint moments are sensitive to unmeasured channels; low observability means many unmeasured-activation patterns produce similar joint moments.
- *M* number of muscles.
- $n_{\mathrm{dof}}$ number of joint degrees of freedom (dimension of the joint-moment vector).
- *r* synergy dimension (rank) in NMF.
- *T* number of time samples.
- $W\in R_{+}^{M\times r}$ synergy weight matrix.
- ${∥\;\cdot \;∥}_{2}$ Euclidean norm.
- λ,γ weights for $J_{2}$ and $J_{3}$ in local refinement.
- $\tau_{\mathrm{ID}}\left(t\right),\tau_{\mathrm{FD}}\left(t\right)$ inverse-dynamics and forward-computed joint moments.

Throughout, we use “activation” to denote the muscle-level control signal (the OpenSim activation state) and reserve “synergy excitation” for the low-dimensional coefficients h(t). In the synergy/EMG literature, activation and excitation are sometimes used interchangeably after preprocessing and normalization; we adopt activation-centric notation for consistency with musculoskeletal simulation conventions. We use “joint moment” as the primary term for τ and refer to the mechanics objective as “joint-moment-consistency”.

### 2.2. Notation and Problem Setup

Let *M* denote the number of muscles and *T* the number of time samples. Let $A\left(t\right)\in {\left[0,1\right]}^{M}$ be the muscle activation vector at time *t*, and define the activation matrix as $A=\left[A\left(1\right),\ldots ,A\left(T\right)\right]\in {\left[0,1\right]}^{M\times T}$. Muscles are partitioned into a measured set $M_{\mathrm{meas}}$ and an unmeasured set $M_{\mathrm{miss}}$, with sizes $M_{\mathrm{meas}}$ and $M_{\mathrm{miss}}$, respectively. Measured activations $A^{\mathrm{meas}}\left(t\right)\in {\left[0,1\right]}^{M_{\mathrm{meas}}}$ are treated as fixed inputs; the unmeasured activations $A^{\mathrm{miss}}\left(t\right)\in {\left[0,1\right]}^{M_{\mathrm{miss}}}$ are decision variables.

Given joint kinematics $\left(q\left(t\right),\dot{q}\left(t\right)\right)$ and inverse-dynamics moments $\tau_{\mathrm{ID}}\left(t\right)$, the forward musculoskeletal model defines a (generally nonlinear) mapping

(1) $$\tau_{\mathrm{FD}}\left(t\right)=F\;\left(A\left(t\right),\;q\left(t\right),\;\dot{q}\left(t\right)\right),$$

where $\tau_{\mathrm{FD}}\left(t\right)$ denotes the net muscle-induced joint moments computed by OpenSim under fixed kinematics (Section 2.6). Both $\tau_{\mathrm{FD}}\left(t\right)$ and $\tau_{\mathrm{ID}}\left(t\right)$ are vectors in $R^{n_{\mathrm{dof}}}$. The inverse problem is used to estimate $A^{\mathrm{miss}}\left(t\right)$, such that $\tau_{\mathrm{FD}}\left(t\right)$ matches $\tau_{\mathrm{ID}}\left(t\right)$ while remaining physiologically plausible.

### 2.3. Algorithm Overview

For clarity, we summarize the proposed reconstruction procedure:

1. NMF: Identify a synergy basis W from proxy activations using NMF (Section 2.4).
2. SynRc: Estimate synergy excitations $\hat{h}\left(t\right)$ from the measured channels via a bounded quadratic program (QP) to obtain a synergy reconstruction prior (SynRc) for unmeasured activations (Section 2.5).
3. Stage I: Approximate a stage-I Pareto set for $\left(J_{1},J_{2}\right)$ over the unmeasured activation time series using derivative-free multi-objective search (Section 2.8).
4. Select trade-off point: Select a stage-I point using the recommended heuristics and use-case guidance (Section 3.6).
5. Stage II: Perform local refinement using a scalarized objective with an additional smoothness penalty (Section 2.8).

**Figure 1:**
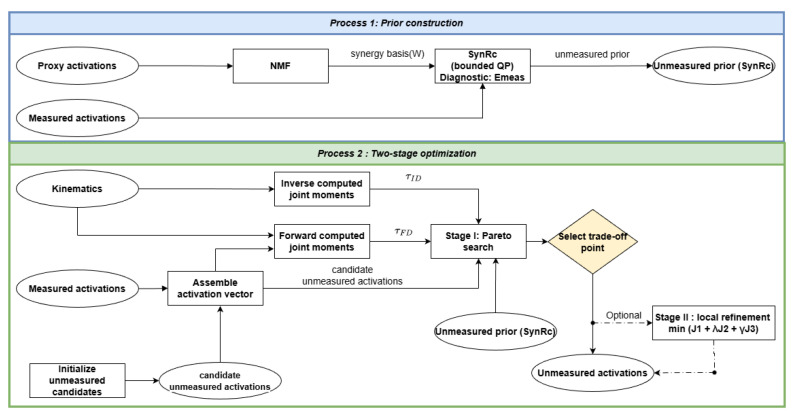
Overview of the proposed workflow. Process 1 constructs a synergy basis W from proxy activations via non-negative matrix factorization (NMF) and computes a SynRc prior from sparse measured channels via a bounded quadratic program (QP); the measured-channel error $E_{\mathrm{meas}}$ serves as a diagnostic of proxy transferability. Process 2 estimates unmeasured activations using a stage-I Pareto search over joint-moment error $J_{1}$ and prior deviation $J_{2}$, followed by an optional stage-II local refinement that minimizes $J_{1}+\lambda J_{2}+\gamma J_{3}$ (Equation (13)).

Figure 1 provides a schematic overview of the two-stage workflow.

### 2.4. Synergy Basis Identification from Proxy Activations

We adopt a standard synergy model where activations are approximated by a low-rank non-negative factorization:

(2) $$A_{\mathrm{proxy}}\approx WH,\;W\in R_{+}^{M\times r},\;H\in R_{+}^{r\times T},$$

where $A_{\mathrm{proxy}}$ is a proxy activation matrix (e.g., from a generic controller or simulated library), W contains synergy weights, and H contains time-varying synergy excitations. The synergy dimension *r* is chosen based on reconstruction quality (e.g., VAF) and interpretability. We compute (W,H) using NMF [20] with non-negativity constraints.

#### Practical Sources of Proxy Activations

In experimental settings, $A_{\mathrm{proxy}}$ is not a “ground truth” signal but a practical device to construct a task-relevant synergy basis W. This practice is motivated by prior observations that synergy structure can be partially shared across healthy subjects and remain relatively stable across repetitions for similar tasks [13,14,15]. Common sources include the following: (i) static optimization (SO) activations computed from the same kinematics and inverse-dynamics moments; (ii) activations from computed muscle control or dynamic optimization on similar tasks; (iii) EMG-derived muscle excitations after preprocessing and calibration (mapped to the activation scale as needed); (iv) a synergy library pooled from prior subjects and tasks in a publicly available dataset or a lab-specific database. The present study focuses on synthetic verification and does not evaluate all proxy sources above; we list them to contextualize how $A_{\mathrm{proxy}}$ may be obtained in experimental workflows. The proxy library can be subject-specific (preferred when available) or generic; in either case, W should be treated as a prior with uncertainty rather than a fixed truth. In practice, SynRc provides an internal sanity check: if the measured channels cannot be reconstructed well from W (high measured-channel error $E_{\mathrm{meas}}$, Equation (5)), then the proxy synergy basis is likely mismatched to the target task/subject (as illustrated in the proxy-mismatch example discussed below), and subsequent stage-I selection should rely more heavily on joint-moment consistency (lower $J_{1}$) or the synergy basis should be updated. In implementation, we apply row-wise normalization of $A_{\mathrm{proxy}}$ (each muscle divided by its maximum over time) to reduce amplitude bias across muscles, perform NMF in the normalized space, and then rescale reconstructions to the original magnitude. To select *r*, we report variance accounted for (VAF) both globally and per muscle:

(3) $${\mathrm{VAF}}_{\mathrm{total}}=1-\frac{\sum_{m=1}^{M}\sum_{t=1}^{T}{\left(A_{mt}-{\left(WH\right)}_{mt}\right)}^{2}}{\sum_{m=1}^{M}\sum_{t=1}^{T}{\left(A_{mt}-{\bar{A}}_{m}\right)}^{2}},$$

where ${\bar{A}}_{m}$ is the mean activation of muscle *m* over time; local VAF is computed analogously for each muscle (row). Unless otherwise stated, VAF is computed in the normalized space (consistent with the NMF fit). Unless otherwise stated, NMF is solved using the alternating least squares algorithm with multiple random initializations, and the best factorization (minimum reconstruction error) is retained.

### 2.5. Synergy Reconstruction Prior from Sparse Observations (SynRc)

Given W and measured activations $A^{\mathrm{meas}}\left(t\right)$, we estimate synergy excitations $\hat{h}\left(t\right)\in {\left[0,1\right]}^{r}$ using a bounded quadratic program. Let $W_{\mathrm{sub}}\in R_{+}^{M_{\mathrm{meas}}\times r}$ be the rows of W corresponding to $M_{\mathrm{meas}}$. For each time step *t*, we solve

(4) $$\begin{array}{c} \hat{h}\left(t\right)=\arg\min_{h(t)}\alpha {∥A^{\mathrm{meas}}\left(t\right)-W_{\mathrm{sub}}h\left(t\right)∥}_{2}^{2}\\ \;+\lambda_{\mathrm{syn}}{∥h(t)∥}_{2}^{2}+\gamma_{\mathrm{syn}}{∥h\left(t\right)-\hat{h}\left(t-1\right)∥}_{2}^{2}\\ s.t.\;0\leq h(t)\leq 1, \end{array}$$

with $\hat{h}\left(0\right)=0$. The first term fits measured activations, the second provides Tikhonov regularization, and the third promotes temporal smoothness in synergy excitation trajectories. Because Equation (4) is a convex bound-constrained quadratic program at each time step, it can be solved efficiently with standard QP solvers (MATLAB R2024b quadprog).

In practice, α and $\lambda_{\mathrm{syn}}$ can be selected by a small grid search that minimizes the measured-channel reconstruction error, while $\gamma_{\mathrm{syn}}$ controls the smoothness of $\hat{h}\left(t\right)$ and can be set based on expected measurement noise. We report the measured-channel error as a diagnostic of proxy-synergy transferability:

(5) $$E_{\mathrm{meas}}=\sqrt{\frac{1}{M_{\mathrm{meas}}T}\sum_{m\in M_{\mathrm{meas}}}\sum_{t=1}^{T}{\left(A_{m}^{\mathrm{meas}}\left(t\right)-{\left(W_{\mathrm{sub}}\hat{h}\left(t\right)\right)}_{m}\right)}^{2}}.$$

The reconstructed full activation prior is

(6) $${\hat{A}}_{\mathrm{SynRc}}\left(t\right)=W\hat{h}\left(t\right),$$

and we extract the unmeasured prior ${\hat{A}}_{\mathrm{SynRc}}^{\mathrm{miss}}\left(t\right)$ for use in the multi-objective problem.

### 2.6. Forward Computation of Muscle-Induced Joint Moments

For a given activation trajectory and fixed kinematics, OpenSim computes musculotendon equilibrium and the associated tendon forces. For each coordinate *k*, the net muscle-induced joint moment can be expressed as

(7) $$\tau_{k}\left(t\right)=\sum_{m=1}^{M}F_{m}\left(t\right)\;r_{m,k}\left(t\right),$$

where $F_{m}\left(t\right)$ is the musculotendon force and $r_{m,k}\left(t\right)$ is the moment arm of muscle *m* about coordinate *k*. Equation (7) includes only muscle-induced contributions; if reserve actuators or other generalized forces are present, their moment contributions should be included in $\tau_{k}\left(t\right)$ when computing $\tau_{\mathrm{FD}}\left(t\right)$. This computation is implemented through the OpenSim Java API by setting $\left(q\left(t\right),\dot{q}\left(t\right)\right)$, assigning activations, equilibrating muscles, and accumulating moment contributions. The underlying musculotendon formulation follows standard Hill-type muscle models as commonly implemented in OpenSim [21]. In the Arm26 benchmark used here, we compute moments for shoulder elevation and elbow flexion ($n_{\mathrm{dof}}=2$). If muscle equilibration fails at a time step (e.g., due to numerical issues), the forward moment evaluation is treated as invalid and the corresponding objective evaluation is penalized, which is important for robust derivative-free optimization.

### 2.7. Multi-Objective Inverse Problem for Unmeasured Activations

We parameterize the unmeasured activations over time as decision variables,

(8) $$x=\mathrm{vec}\;\left(A^{\mathrm{miss}}\right)\in R^{n},\;n=M_{\mathrm{miss}}T,$$

with bound constraints $0\leq x_{i}\leq 1$. Measured activations are held fixed to their observed values, and x fills only the unmeasured channels.

#### Why a Second-Stage Optimization?

SynRc yields a physiologically structured prior that is consistent with measured channels and a low-dimensional synergy model, but it does not enforce that the resulting full activations reproduce inverse-dynamics joint moments through the nonlinear forward map F in Equation (1). The second stage explicitly enforces joint-moment consistency and quantifies the trade-off between mechanical fidelity and neural consistency. When inverse dynamics are unreliable or only a fast prior-only estimate is needed, the SynRc-only baseline provides a lightweight reconstruction that does not enforce joint-moment consistency by design. Mechanically sensitive studies benefit from the full multi-objective stage (torque-comparison results).

We define the following objectives:

1. **Joint-moment-consistency objective:**
  (9) $$J_{1}\left(x\right)=\frac{\sqrt{\sum_{t=1}^{T}{∥\tau_{\mathrm{FD}}\left(t;x\right)-\tau_{\mathrm{ID}}\left(t\right)∥}_{2}^{2}}}{\sqrt{\sum_{t=1}^{T}{∥\tau_{\mathrm{ID}}\left(t\right)∥}_{2}^{2}}}.$$
  Normalization makes $J_{1}$ scale-invariant across tasks and facilitates comparison across Pareto solutions.
  *Interpretation note:* $J_{1}$ compares the inverse-dynamics target $\tau_{\mathrm{ID}}\left(t\right)$, which represents net joint moments, against the muscle-induced forward moments $\tau_{\mathrm{FD}}\left(t\right)$ produced by OpenSim under fixed kinematics. Therefore, even the synthetic computed muscle control (CMC) reference is not guaranteed to minimize $J_{1}$, and local refinement can achieve a lower $J_{1}$ by fitting residual mismatch between $\tau_{\mathrm{ID}}$ and $\tau_{\mathrm{FD}}$. We accordingly interpret $J_{1}$ as a joint-moment-consistency diagnostic rather than a guarantee of physiological correctness.
2. **Neural-consistency objective (deviation from SynRc prior):**
  (10) $$J_{2}\left(x\right)=\sqrt{\frac{1}{M_{\mathrm{miss}}T}\sum_{m\in M_{\mathrm{miss}}}\sum_{t=1}^{T}{\left(A_{m}^{\mathrm{miss}}\left(t;x\right)-{\hat{A}}_{\mathrm{SynRc},m}^{\mathrm{miss}}\left(t\right)\right)}^{2}}.$$
3. **Smoothness penalty** (used in local refinement):
  (11) $$J_{3}\left(x\right)=\sqrt{\frac{1}{M_{\mathrm{miss}}\left(T-1\right)}\sum_{m\in M_{\mathrm{miss}}}\sum_{t=1}^{T-1}{\left(A_{m}^{\mathrm{miss}}\left(t+1;x\right)-A_{m}^{\mathrm{miss}}\left(t;x\right)\right)}^{2}}.$$

### 2.8. Two-Stage Solver: Pareto Front Search and Local Refinement

The primary trade-off is between $J_{1}$ and $J_{2}$, so we first approximate the Pareto front for $\min(J_{1},J_{2})$ under bound constraints using a derivative-free direct search method [22], and report the resulting non-dominated output as a stage-I Pareto set. Initial points include the SynRc prior ${\hat{A}}_{\mathrm{SynRc}}^{\mathrm{miss}}$ and additional random feasible points to encourage exploration. As a reproducible default when no strong preference is available, we select the stage-I point using the utopia-closest criterion on min–max normalized objectives (Equation (12)); task-dependent alternatives are discussed in Section 3.6. Specifically, we select

(12) $$k^{★}=\arg\min_{k\in \{1,\ldots ,K\}}{∥\left[\begin{array}{c} {\tilde{J}}_{1}\left(k\right)\\ {\tilde{J}}_{2}\left(k\right) \end{array}\right]∥}_{2},\;{\tilde{J}}_{i}\left(k\right)=\frac{J_{i}\left(k\right)-\min_{j}J_{i}\left(j\right)}{\max_{j}J_{i}\left(j\right)-\min_{j}J_{i}\left(j\right)}.$$

After selecting a point from the stage-I Pareto set based on task requirements, we perform local refinement with a scalarized objective that includes smoothness:

(13) $$J\left(x\right)=J_{1}\left(x\right)+\lambda \;J_{2}\left(x\right)+\gamma \;J_{3}\left(x\right),$$

where λ is derived from the local slope of the stage-I Pareto set in the $\left(J_{1},J_{2}\right)$ plane and γ≥0 is a user-specified smoothness weight. In practice, given a selected stage-I index $k^{★}$, we approximate

(14) $$\lambda \approx \frac{J_{1}\left(k^{★}+1\right)-J_{1}\left(k^{★}-1\right)}{J_{2}\left(k^{★}-1\right)-J_{2}\left(k^{★}+1\right)},$$

using neighboring stage-I points (with appropriate boundary handling). The Pareto stage and local refinement are implemented with MATLAB paretosearch and patternsearch, respectively. In our implementation, we target 30 non-dominated solutions and use a function-evaluation budget of 90,000; objective evaluations are parallelized when available. Additional solver settings are available from the corresponding author upon reasonable request (within the scope necessary to reproduce the reported results). Here, ParetoSetSize specifies a target number of non-dominated solutions to return; the resulting set is an approximation under finite evaluation budgets and mesh tolerances. Accordingly, we refer to the output as a *stage-I Pareto set* (an approximate trade-off set) rather than the true Pareto front, and we report selection sensitivity by inspecting neighboring points and, when refinement is used, a small γ sweep.

### 2.9. Synthetic Benchmark Design and Implementation Details

We verify the method on a synthetic OpenSim arm benchmark (Arm26) [23] with two degrees of freedom (shoulder elevation and elbow flexion) and six muscles. Ground-truth kinematics and full muscle activations are generated using OpenSim computed muscle control (CMC) [24,25] and treated as the reference solution. Inverse-dynamics moments are computed from the same kinematics using OpenSim’s inverse dynamics tool [1,2]. To emulate typical preprocessing of experimental inverse dynamics, we filter the ID moment trajectories using a zero-phase 4th-order Butterworth low-pass filter with a 5 Hz cutoff (MATLAB butter/filtfilt). The filtered ID moments are then interpolated to the activation time grid for objective evaluation. We emulate sparse sensing by treating four muscles as measured—triceps long head (TRIlong), triceps medial head (TRImed), biceps long head (BIClong), and brachialis (BRA)—and two muscles as unmeasured—triceps lateral head (TRIlat) and biceps short head (BICshort). The measured activation channels are taken from the CMC reference and treated as the “observed” signals in this synthetic verification.

Synergies are identified via NMF with r=4 from proxy activations. For SynRc, we set $\gamma_{\mathrm{syn}}=0.1$ for temporal regularization and selected α=10 and $\lambda_{\mathrm{syn}}=0.01$ using a small grid search that minimizes measured-channel reconstruction error. In this verification study, the proxy activations are derived from the same synthetic pipeline, enabling controlled analysis with known ground truth while also representing an optimistic scenario in which synergy structure is well matched to the target task. The full dataset contains T=528 time samples spanning 0.71 s. All computations are performed in MATLAB (R2024b) with the OpenSim Java API (v4.x). The OpenSim model files used in this study are publicly available from the OpenSim official website. The scripts and derived numerical results required to reproduce the benchmark are available from the corresponding author upon reasonable request (within the scope necessary for academic use and reproduction of the reported results). Solver settings and hyperparameters are summarized in Table 1.

#### 2.9.1. Case 2: Alternative Measured Set(Setup)

We repeat the Arm26 benchmark under an alternative measured set while retaining the same model and motion. Specifically, we treat TRIlong, TRIlat, BIClong, and BICshort as measured and reconstruct TRImed and BRA as unmeasured. Results are reported in Section 3.2.

#### 2.9.2. Case 3: Different Model and Task(Setup) (gait10dof18musc Walking)

To demonstrate portability beyond Arm26, we consider a different musculoskeletal model and motion. We use OpenSim’s distribution test asset gait10dof18musc_subject01 with a walking inverse-kinematics trajectory. Because this minimal asset does not include a full CMC/ID pipeline, we construct a fully synthetic benchmark with known ground truth. We generate smooth ground-truth activations for a right-leg muscle subset (hamstrings_r, rect_fem_r, vasti_r, gastroc_r, soleus_r, tib_ant_r). We treat rect_fem_r, vasti_r, soleus_r, and tib_ant_r as measured, and reconstruct hamstrings_r and gastroc_r as unmeasured. To form the mechanical objective, we precompute OpenSim moment arms for these muscles about hip flexion, knee angle, and ankle angle along the walking kinematics, and use a lightweight forward moment map $\tau_{\mathrm{FD},k}\left(t\right)=-\sum_{m}r_{m,k}\left(t\right)\;F_{m}^{\max}\;a_{m}\left(t\right)$ (for each coordinate *k*), with synthetic targets $\tau_{\mathrm{ID}}\left(t\right)$ generated from the ground truth. This Case 3 forward model is intentionally a *linearized* approximation: it uses OpenSim geometry (time-varying moment arms $r_{m,k}\left(t\right)$) but replaces the full activation-to-force dynamics with a static proportional model using $F_{m}^{\max}a_{m}\left(t\right)$, thereby ignoring force–length/velocity effects, tendon compliance, passive forces, and activation dynamics. Accordingly, Case 3 is intended as a minimal cross-model demonstration that the Pareto/selection machinery remains meaningful under different musculoskeletal geometry, rather than as a quantitative gait prediction study. To avoid a degenerate trade-off in this small muscle subset, we additionally scale the maximum isometric force of the unmeasured muscles by a factor of 2 (a stress-test analogous to the observability scaling in Section 3.5, not a calibrated subject-specific parameter). Because $\tau_{\mathrm{ID}}$ is generated from the same forward map, this case does not probe ID–forward mismatch; ID–forward mismatch diagnostics are instead characterized by the Arm26 robustness tests in Section 3.4. Synergies are identified from separate proxy activations, and the SynRc prior is computed from the measured channels as in the Arm26 benchmark. Results are reported in Section 3.3.

### 2.10. Evaluation Metrics and Baselines

Because the benchmark is synthetic, ground-truth activations are known for all muscles. We evaluate reconstruction accuracy for each unmeasured muscle using the time-domain root-mean-square error (RMSE) between the estimate and the ground truth. To characterize mechanical consistency, we also report the normalized joint-moment error $J_{1}$ (Section 2.7) and visualize the stage-I Pareto set over $\left(J_{1},J_{2}\right)$, where $J_{2}$ measures deviation from the SynRc prior. We compare the following:

- **SO baseline:** OpenSim static optimization activations [1,3].
- **SynRc-only baseline:** The SynRc prior for the unmeasured activations (no joint-moment-consistency optimization).
- **Joint-moment-only + smoothness baseline:** A joint-moment-tracking reconstruction without a synergy prior, minimizing $J_{1}+\gamma J_{3}$ under a reduced-dimension parameterization.
- **Proposed method:** Two-stage multi-objective optimization with different stage-I selections and smoothness weights γ.

These baselines are not intended as a comprehensive comparison with state-of-the-art activation estimation pipelines. Rather, they isolate the effects of a synergy-based prior (SynRc-only) and mechanics-based fitting (SO and joint-moment-only) so that the paper’s main claim—making mechanics–synergy trade-offs explicit and diagnosable via Pareto geometry and selection sensitivity—can be evaluated without relying on a claim of superior reconstruction accuracy.

## 3. Results

### 3.1. Synergy Identification and SynRc Prior Quality

**Figure 2:**
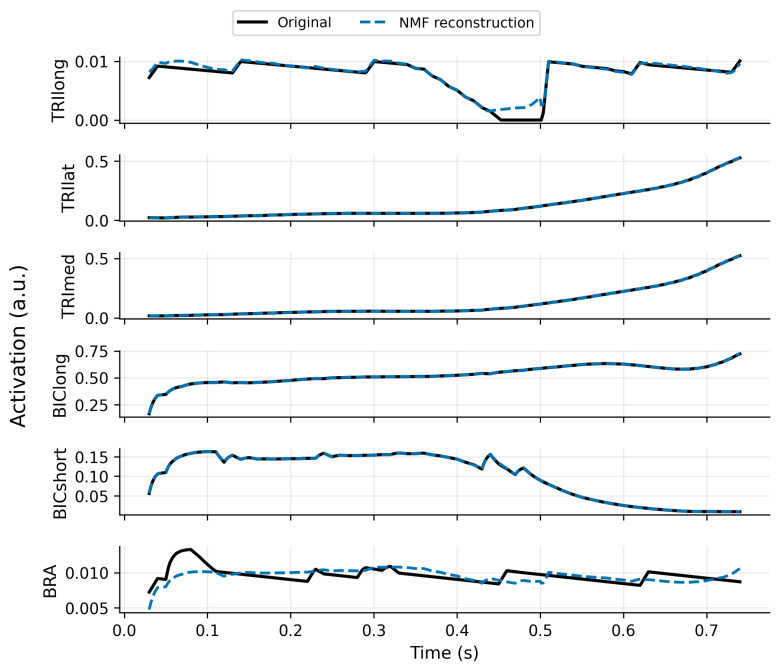
NMF with r=4 captures the proxy activations with high fidelity while preserving structure. Curves compare proxy activations and their NMF reconstructions for representative muscles (shown on the rescaled activation axis).

**Figure 3:**
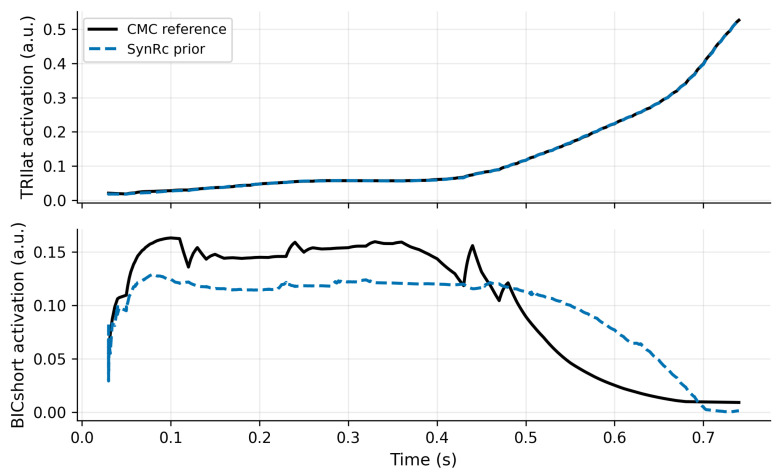
SynRc reconstructs TRIlat well but yields larger error for BICshort in this benchmark. Solid: CMC reference; dashed: SynRc prior for the unmeasured muscles (TRIlat, BICshort), with RMSE reported in the caption text.

For r=4, NMF yields a high global reconstruction quality (total VAF =99.4%) while preserving interpretability; local VAF remains high for most muscles but is lower for BRA (54.96%), indicating that this muscle is less well represented by the chosen synergy dimension. Figure 2 illustrates NMF reconstruction versus proxy activations. SynRc reconstructs TRIlat with very low RMSE (≈$9.4\times 10^{-4}$) but shows larger errors for BICshort (RMSE ≈0.033), consistent with limited observability from the measured subset (Figure 3).

#### Robustness to Proxy-Synergy Mismatch

**Figure 4:**
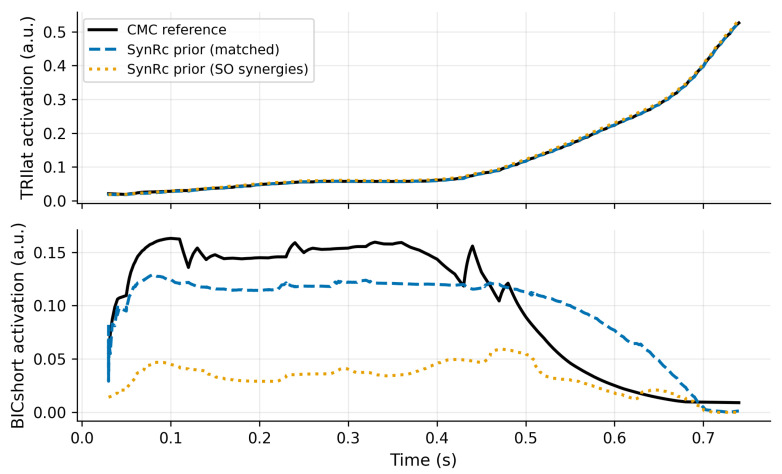
Proxy-synergy mismatch increases prior error for unmeasured muscles even when measured channels fit well. We compare SynRc priors built from a matched proxy library versus SO-derived synergies; $E_{\mathrm{meas}}$ (Equation (5)) provides a measured-only proxy-transfer diagnostic.

The preceding analysis uses proxy synergies derived from the same synthetic pipeline as the target motion (an optimistic setting). To emulate a mismatch between the proxy synergy library and the target task, we identify the synergy basis from static-optimization (SO) activations and then recompute the SynRc prior using the same measured channels from the CMC reference. Figure 4 shows that a mismatch in the proxy basis can substantially degrade prior accuracy for the unmeasured muscles (most strongly for BICshort), even though the measured channels remain well reconstructed. Quantitatively, the measured-channel RMSE increases from ≈$1.7\times 10^{-3}$ (matched proxy) to ≈$2.8\times 10^{-3}$ (SO proxy), while unmeasured-muscle RMSE increases from ≈$9.4\times 10^{-4}$ to ≈$3.7\times 10^{-3}$ for TRIlat and from ≈$3.3\times 10^{-2}$ to ≈$8.4\times 10^{-2}$ for BICshort. This suggests a practical diagnostic in applications without ground truth: the measured-channel reconstruction error of SynRc provides an internal check on whether the proxy synergy basis is transferable, and can inform how strongly $J_{2}$ should be trusted relative to $J_{1}$ during stage-I selection.

### 3.2. Case 2: Alternative Measured Set

**Figure 5:**
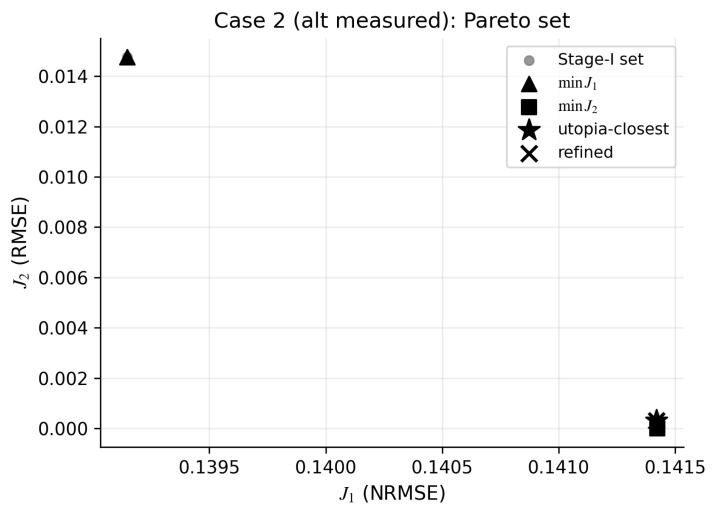
Case 2 shows a near-degenerate $\left(J_{1},J_{2}\right)$ geometry, so refinement changes little beyond SynRc. the plot shows the stage-I Pareto set (joint-moment error $J_{1}$ versus prior deviation $J_{2}$), the utopia-closest point (star), and the refined solution (cross). Measured: TRIlong, TRIlat, BIClong, BICshort; unmeasured: TRImed, BRA. Mechanically dominant corresponds to lower $J_{1}$; neurally dominant corresponds to lower $J_{2}$.

We next evaluate the Case 2 configuration with an alternative measured set (Section 2.9.1). For this configuration, the SynRc diagnostic remains low ($E_{\mathrm{meas}}\approx 1.5\times 10^{-3}$), and the stage-I trade-off set collapses to a narrow band, indicating that the two objectives are largely aligned for this motion and measured set. Figure 5 and Table 2 show that the utopia-closest selection coincides with the SynRc prior and that stage-II refinement produces only negligible changes, illustrating how the framework can diagnose when additional joint-moment-driven optimization provides little benefit beyond the synergy-based reconstruction.

### 3.3. Case 3: Different Model and Task (gait10dof18musc Walking)

**Figure 6:**
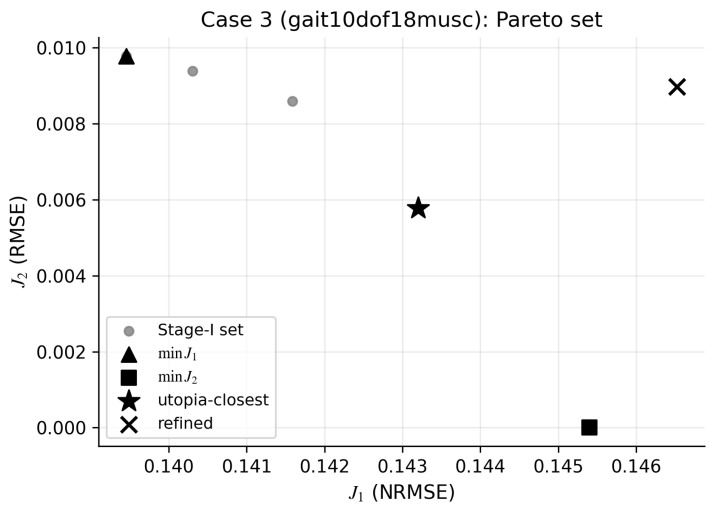
Case 3 shows a non-degenerate $\left(J_{1},J_{2}\right)$ trade-off under a different model/task. Plot shows the stage-I Pareto set, the utopia-closest point (star), and the refined solution (cross). Measured: rect_fem_r, vasti_r, soleus_r, tib_ant_r; unmeasured: hamstrings_r, gastroc_r. Mechanically dominant corresponds to lower $J_{1}$; neurally dominant corresponds to lower $J_{2}$.

Case 3 evaluates the method on a different model and task (Section 2.9.2). Figure 6 shows a nontrivial $\left(J_{1},J_{2}\right)$ geometry under this different model and task, and Table 3 summarizes representative solutions and unmeasured-muscle RMSE against the known ground truth.

### 3.4. Synthetic Robustness Tests Under Noise and Mismatch

The preceding synthetic benchmark is optimistic because the measured activations, proxy synergies, and inverse-dynamics moments are generated within a consistent OpenSim pipeline. To better characterize when the proposed selection rules are reliable (and when they can fail), we perform four synthetic robustness tests by introducing controlled perturbations to (i) inverse-dynamics (ID) moments, (ii) measured activation channels, (iii) proxy synergy mismatch, and (iv) forward-model parameter mismatch. Because re-running the full Pareto search for every perturbation is computationally expensive, we adopt a post-hoc protocol: we keep the baseline stage-I Pareto decision set ${\left\{x\left(k\right)\right\}}_{k=1}^{K}$ fixed (returned by paretosearch, K=30) and re-evaluate objective values under each perturbation to assess the stability of the $\left(J_{1},J_{2}\right)$ trade-off geometry and the resulting point selection. For a deterministic representative choice, we apply the same utopia-closest rule (Equation (12)) on the perturbed objectives and report the selected index $k^{★}$. This protocol evaluates selection sensitivity *within* a fixed candidate set and should be interpreted as a robustness diagnostic rather than a re-computation of the true Pareto front under perturbation.

Supplementary Figure S1 summarizes the results. ID perturbations include additive Gaussian noise (5% of the root mean square (RMS) of each degree-of-freedom moment) and a small phase bias (5 ms) applied to the ID target. Measured-channel perturbations include per-channel gain error (±10%) and additive noise (standard deviation 0.02 in activation units, followed by clipping to [0,1]); this directly increases the measured-channel SynRc reconstruction error $E_{\mathrm{meas}}$ and can degrade the proxy-based prior. Finally, proxy-synergy mismatch is graded by mixing the synergy basis with a random basis (mismatch level δ∈{0,0.1,0.2,0.3}), which monotonically increases both $E_{\mathrm{meas}}$ and the unmeasured-muscle prior RMSE. To emulate a common source of musculoskeletal model uncertainty, we also perturb forward-model muscle strength by scaling each muscle’s maximum isometric force by a random factor in [1−0.1,1+0.1] and recompute forward moments under the perturbed model. Perturbation magnitudes are chosen to be moderate on the normalized scales of this benchmark and are intended as stress-tests rather than as calibrated experimental noise models. Supplementary Table S1 reports the corresponding objective values and unmeasured-muscle RMSE at the selected point. To account for stochastic variability, we repeat the ID-noise and proxy-mismatch perturbations over 20 random seeds, and we repeat the measured-channel perturbation and the model-parameter mismatch over 5 random seeds. Supplementary Table S2 reports mean ± SD statistics at the selected point and the empirical range of the selected index $k^{★}$. Overall, these tests highlight that (a) even modest ID bias can substantially alter $J_{1}$ (and thus, the selected point), and (b) increases in $E_{\mathrm{meas}}$ track degradation of the synergy prior under measured-signal noise and proxy mismatch, motivating a shift toward $J_{1}$-dominant selections when $E_{\mathrm{meas}}$ is high.

### 3.5. Multi-Case Synthetic Study: Observability Regimes

**Figure 7:**
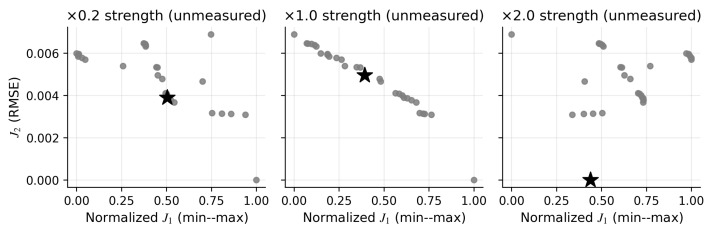
Weak unmeasured muscles flatten the joint-moment error spread within the stage-I set. We scale the strength of unmeasured muscles (TRIlat, BICshort) and re-evaluate $\left(J_{1},J_{2}\right)$ on a fixed stage-I candidate set; gray dots denote the stage-I candidate solutions and stars denote utopia-closest selections across regimes.

Method behavior depends on the degree to which the mechanical objective is informative about the unmeasured channels. To provide a small set of representative synthetic cases (beyond a single benchmark), we construct three “observability regimes” by scaling the maximum isometric force of the unmeasured muscles (TRIlat and BICshort) while keeping the measured channels and kinematics fixed. Intuitively, when unmeasured muscles are weak, net joint moments become less sensitive to their activations, producing a flatter trade-off geometry; when unmeasured muscles are strong, the sensitivity increases and the Pareto set becomes more structured. For each regime, we keep the baseline stage-I decision set fixed and re-evaluate $J_{1}$ under the modified forward model, while $J_{2}$ (deviation from the SynRc prior) is unchanged by construction. Figure 7 shows that the resulting $\left(J_{1},J_{2}\right)$ geometry and the utopia-closest selection can change substantially across regimes, highlighting that a narrow $J_{1}$ spread is not a solver failure but a diagnostic of limited mechanical observability under sparse measurements. Table 4 reports the selected index and the empirical $J_{1}$ range within the candidate set for each regime.

### 3.6. Pareto Front and Task-Dependent Solution Selection

The multi-objective formulation yields a Pareto front over $\left(J_{1},J_{2}\right)$. How to read the Pareto plot: each point is a candidate reconstruction in the stage-I set. Moving toward lower $J_{1}$ improves joint-moment consistency (mechanically dominant), whereas moving toward lower $J_{2}$ increases neural consistency by staying closer to the synergy prior (neurally dominant). In applications, the stage-I point can be selected based on task-specific confidence in ID moments versus synergy priors. We report representative cases where the stage-I point and the smoothness weight γ are varied to illustrate the trade-offs. For this synthetic dataset, the stage-I Pareto set returned by paretosearch spans a narrow range of joint-moment errors ($J_{1}\approx 0.1533$ to 0.1539) but a broader range of neural deviations ($J_{2}\approx 0$ to $6.9\times 10^{-3}$). Within this explored stage-I set, the dominant trade-off is thus in closeness to the SynRc prior while producing only modest changes in net joint moments. This narrow $J_{1}$ spread reflects the redundancy of the benchmark: for this motion and measured set, the net joint moments are relatively insensitive to variations in the unmeasured channels within the explored region, so many distinct activation patterns yield similar moments. We interpret stage-I point 1 as $J_{1}$-dominant (minimum $J_{1}$ on the stage-I set, largest $J_{2}$), stage-I point 30 as neural-dominant (minimum $J_{2}$), and stage-I point 18 as an intermediate stage-I choice used in the subsequent reconstruction tests. Because the Pareto stage is an approximation under finite budgets and mesh tolerances, and because stage-II refinement minimizes a different scalarized objective that also includes $J_{3}$, the refined solution is not guaranteed to remain on the stage-I Pareto set in $\left(J_{1},J_{2}\right)$. In particular, $J_{1}$-dominant selections can move away from the stage-I Pareto set while reducing $J_{1}$ beyond the minimum observed in stage I (Table 5).

#### 3.6.1. Neighborhood Sensitivity

To quantify local sensitivity around the utopia-closest selection, Supplementary Table S4 reports objectives and unmeasured-muscle RMSE for $k^{★}$ and a small index neighborhood ($k^{★}\pm 2$) on the stage-I set. In this Arm26 benchmark, the neighborhood shows small $J_{1}$ variation but non-negligible changes in smoothness ($J_{3}$) and BICshort RMSE, so reporting Δk sensitivity provides a simple robustness check when ground truth is unavailable.

#### 3.6.2. Scalarization Baseline

**Figure 8:**
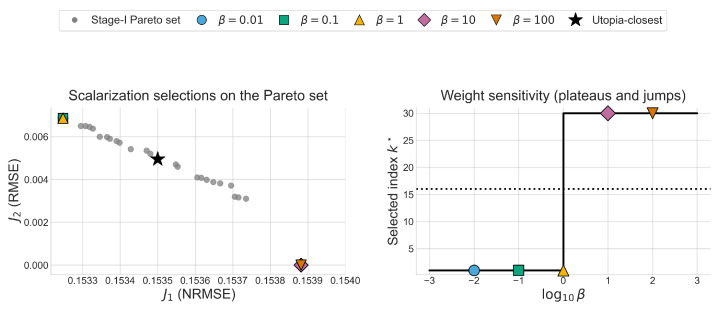
Weighted-sum scalarization selects only a few discrete Pareto points as β varies. (**Left**): stage-I set with representative β-selected points and the utopia-closest point (star); (**right**): selected index $k_{\beta }^{★}$ versus β after min–max normalization of $J_{1}$ and $J_{2}$.

A common alternative to explicit Pareto analysis is to scalarize objectives with a fixed weighted sum. To provide a simple baseline, we apply a weighted-sum rule on the stage-I Pareto set: $k_{\beta }=\arg\min_{k}\left({\tilde{J}}_{1}\left(k\right)+\beta \;{\tilde{J}}_{2}\left(k\right)\right)$, where β>0 is a weight ratio and ${\tilde{J}}_{i}$ are min–max normalized objectives over the stage-I set (same normalization as Equation (12)). Figure 8 shows that, even after normalization, the selected index can be piecewise constant over wide ranges of β and can jump abruptly as β changes, effectively selecting only a few discrete solutions. This sensitivity makes weight selection difficult to justify a priori and motivates reporting the full trade-off set and neighborhood sensitivity rather than committing to a single fixed scalarization.

**Figure 9:**
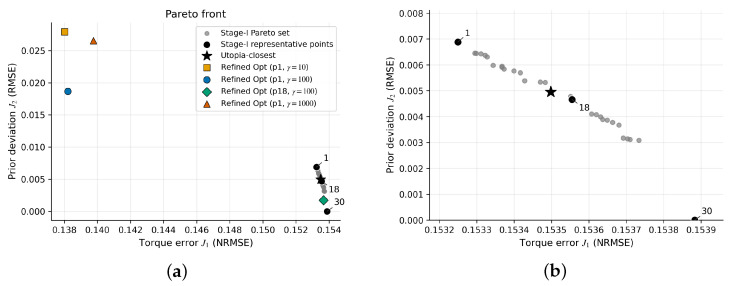
Stage-II refinement can move off the stage-I $\left(J_{1},J_{2}\right)$ set while reducing $J_{1}$. Plot shows the stage-I Pareto set (joint-moment error $J_{1}$ versus prior deviation $J_{2}$), the utopia-closest point (star; Equation (12)), and refined test solutions (cross). (**a**) Full view including refined solutions; (**b**) zoom-in near the stage-I set. Mechanically dominant corresponds to lower $J_{1}$; neurally dominant corresponds to lower $J_{2}$.

Figure 9 shows the stage-I $\left(J_{1},J_{2}\right)$ set, the utopia-closest point (star; Equation (12)), and representative refined solutions (cross).

### 3.7. Unmeasured Activation Reconstruction Results

Figure 5 and Figure 6 illustrate two representative trade-off geometries (near-degenerate versus non-degenerate) to clarify when the proposed Pareto machinery is informative; Figure 9 reports the main-case trade-off and refined off-Pareto behavior.

**Figure 10:**
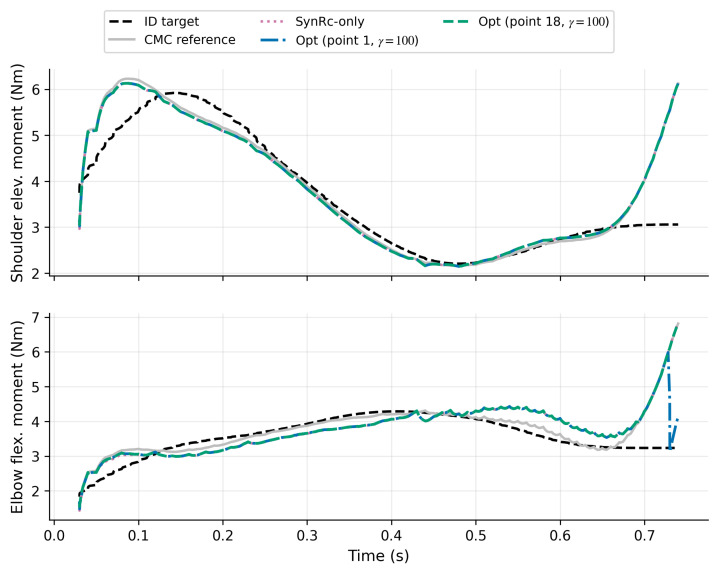
Joint-moment fitting mainly changes the elbow moment near the terminal phase. Curves compare $\tau_{\mathrm{ID}}$ (target) and forward-computed $\tau_{\mathrm{FD}}$ under fixed kinematics for CMC, SynRc-only, and representative optimized solutions (stage-I point and γ); top: shoulder elevation, bottom: elbow flexion.

Table 6 summarizes RMSE for unmeasured muscles under SO, SynRc-only (prior-only; no joint-moment-consistency optimization), a joint-moment-only baseline with smoothness regularization, and the proposed method with four configurations (stage-I point and γ). Relative to SO, the synergy-based prior (SynRc-only) reduces BICshort RMSE by ≈21%; the multi-objective stage primarily improves joint-moment tracking and influences TRIlat depending on the stage-I point and γ. The joint-moment-only baseline improves joint-moment tracking compared with SynRc-only (Table 5) but yields TRIlat reconstruction that is substantially less accurate than the synergy-driven prior, illustrating the role of $J_{2}$ in preserving neural-consistent structure when joint-moment information alone is insufficient to uniquely determine unmeasured activations. In this benchmark, BICshort remains close to the SynRc prior across configurations, whereas TRIlat is more sensitive to the stage-I selection; Supplementary Figure S3 provides a brief sensitivity check for the refinement smoothness weight γ. To connect activation reconstruction to the multi-objective formulation, Table 5 reports the corresponding joint-moment error $J_{1}$, prior deviation $J_{2}$, and smoothness $J_{3}$ for the synthetic reference (CMC), the SynRc-only baseline (direct synergy reconstruction without joint-moment-consistency optimization), a joint-moment-only smoothness-regularized baseline, and the optimized solutions (Opt). $J_{1}$-dominant selections (stage-I point 1) reduce $J_{1}$ from 0.154 (SynRc-only) and 0.141 (CMC) to ≈0.138, but increase deviation from the SynRc prior ($J_{2}\approx 1.9\times 10^{-2}$ to $2.8\times 10^{-2}$) and can degrade TRIlat accuracy. Conversely, the more neural-consistent selection (stage-I point 18) keeps $J_{2}$ close to zero and yields near-ground-truth TRIlat reconstruction, at the expense of a larger $J_{1}$ comparable to the synergy baseline. Notably, even the synthetic CMC reference has nonzero $J_{1}$ because inverse-dynamics moments represent net joint moments and are not, in general, identical to muscle-induced moments computed by the forward model under fixed kinematics. Figure 10 visualizes the resulting shoulder and elbow moments across representative solutions, highlighting how $J_{1}$-dominant refinement primarily alters the elbow-flexion moment near the terminal phase to reduce mismatch with the inverse-dynamics target. Supplementary Figure S2 further compares reconstructed unmeasured activations across the four representative optimized configurations, illustrating how stage-I selection and γ jointly shape TRIlat and BICshort trajectories relative to SO and ground truth.

## 4. Discussion

The results highlight several practical implications of synergy-informed, joint-moment-consistent reconstruction under sparse measurements. In the Arm26 benchmark, optimized solutions reduce $J_{1}$ from 0.154 (SynRc-only) to ≈0.138. More neural-consistent selections achieve TRIlat RMSE $\approx 10^{-3}$ (Table 5 and Table 6). First, synergy reconstruction can be highly accurate for some muscles (TRIlat in this benchmark). However, the synergy prior alone does not guarantee joint-moment consistency. Enforcing joint-moment matching through the forward model reconciles neural priors with physics and makes the trade-off explicit via the stage-I Pareto set. Second, solution quality is sensitive to stage-I point selection. A $J_{1}$-dominant point can degrade agreement with ground-truth activations even when the synergy prior is accurate, whereas a more neural-consistent point can recover near-ground-truth activations. This supports task-dependent selection rules based on relative confidence in inverse dynamics moments, model fidelity, and the transferability of synergy structure. Third, the smoothness weight γ acts as a user-specified sensitivity parameter in local refinement. Increasing γ reduces temporal fluctuations but can oversmooth and degrade accuracy. We therefore recommend reporting a small γ sweep (Supplementary Figure S3) and choosing γ in relation to sampling density and expected measurement noise, rather than treating it as a single fixed choice. Finally, the observability-regime cases show that the stage-I geometry can diagnose identifiability under sparse measurements. A very narrow $J_{1}$ spread indicates that joint-moment information is weakly informative about unmeasured channels within the explored region. In such regimes, selection should rely more on prior confidence and measured-channel diagnostics than on small differences in $J_{1}$.

### 4.1. Selection Workflow Without Ground Truth

For applications without ground truth, we suggest reporting the following checklist to document diagnostics and selection choices. This checklist is intended as a reporting aid rather than a validated default pipeline:

1. Report the stage-I Pareto set ${\left\{\left(J_{1}\left(k\right),J_{2}\left(k\right)\right)\right\}}_{k=1}^{K}$ and the SynRc diagnostic $E_{\mathrm{meas}}$ (Equation (5)) as a proxy-transfer indicator;
2. Report ID-target quality diagnostics (e.g., high-frequency ratio, spike score, estimated lag under synchronization checks, and the minimum achievable $J_{1}$ within the stage-I set) to contextualize how strongly $J_{1}$ should be trusted;
3. State the selection rationale (e.g., endpoint toward low $J_{1}$ or low $J_{2}$, or a compromise rule such as utopia-closest) and report the selected index $k^{★}$ and $\left(J_{1}\left(k^{★}\right),J_{2}\left(k^{★}\right)\right)$;
4. Report sensitivity by evaluating a small neighborhood on the stage-I set and, when refinement is used, a small γ sweep.

To make this procedure reproducible and less subjective, we recommend reporting a small, standardized “protocol summary” in every experiment (Table 7). Table 7 is designed as a fill-in template. Report each diagnostic, state the selection rule, and document neighborhood/γ sensitivity. An objective default for the compromise option in Step 3 is the utopia-closest stage-I point using min–max normalized objectives (Equation (12)). When the empirical range of one objective is very narrow (e.g., $J_{1}$ in highly redundant or weakly observable regimes), min–max normalization can amplify small numerical differences. We therefore recommend interpreting $k^{★}$ together with neighborhood sensitivity (Supplementary Table S4) rather than as a definitive optimum.

### 4.2. Relation to Prior Work

Synergy extrapolation and related methods infer unmeasured activations from measured channels by exploiting low-dimensional structure [16,17,18,26]. Our formulation is complementary to these approaches. We treat the synergy-derived estimate (SynRc) as an explicit prior. Rather than committing to a single synergy-driven reconstruction, we place this prior in competition with the joint-moment-consistency objective $J_{1}$ computed from the musculoskeletal forward model.

This separation makes the trade-off transparent (via $\left(J_{1},J_{2}\right)$) and avoids selecting fixed scalarization weights a priori. Compared with EMG-driven and hybrid approaches [7,8,9], the present study focuses on sparse-activation reconstruction under fixed kinematics. We do not model excitation-to-activation dynamics or perform EMG-driven parameter calibration. Integrating Pareto-based selection with EMG-driven pipelines (where measured channels are EMG-derived excitations) is a natural extension.

From an experimental perspective, EMG-based validation itself involves a spatial-resolution trade-off: surface EMG is noninvasive and practical but can be limited for deep or closely spaced muscles, whereas needle or fine-wire EMG provides higher spatial specificity at the cost of participant discomfort and constraints on natural movement. This trade-off further motivates sparse-measurement scenarios in which only a subset of muscles can be reliably instrumented.

### 4.3. Limitations and Future Directions

This study is a verification on synthetic data with known ground truth [19]. Ground-truth activations, kinematics, and inverse dynamics are all generated within a consistent OpenSim pipeline, so the benchmark can underestimate experimental uncertainty—particularly noise, soft-tissue artifacts, force-plate errors, and musculoskeletal parameter uncertainty. We partially address these concerns through synthetic stress-tests that perturb ID targets, measured-channel noise, proxy-synergy structure, and model parameters (Section 3.4), though these perturbations are not intended to fully replicate experimental conditions.

A further caveat concerns the inverse-dynamics targets themselves. The moments used as $J_{1}$ targets are net joint moments, which are not purely muscle-induced; even the CMC reference exhibits residual mismatch. $J_{1}$-dominant optimization can exploit this mismatch by introducing sharp activation changes near motion endpoints. Mitigating such effects will require additional physiological constraints (e.g., activation dynamics and bounds on activation rate) and validation under calibrated experimental noise and modeling errors.

The Arm26 model is intentionally small (2 degrees of freedom, 6 muscles) and should be viewed as a controlled proof-of-concept. Establishing practical utility will require evaluation on larger models with stronger redundancy (e.g., lower-limb gait models with dozens of muscles). Case 3 provides a cross-model demonstration. However, it uses a linearized forward-moment map and a deliberate unmeasured $F^{\max}$ scaling stress-test, and it generates $\tau_{\mathrm{ID}}$ from the same simplified map. Therefore, Case 3 should be interpreted as evidence that the Pareto/selection framework transfers to different musculoskeletal geometry, not as a validation of full gait dynamics or experimental inverse dynamics. We also provide a sensitivity analysis using SO-derived synergies. Broader mismatch across tasks/subjects remains important future work.

Finally, this paper does not include a head-to-head numerical comparison with alternative approaches (e.g., EMG-driven synergy extrapolation pipelines or full optimal-control formulations) because the scope is limited to synthetic verification. Such comparisons are an important next step once appropriate experimental datasets are available.

### 4.4. Computational Considerations

Computational cost is a major practical consideration because each objective evaluation requires OpenSim muscle equilibration across *T* time samples. Accordingly, the present implementation is intended for offline analyses where interpretability and mechanical fidelity justify longer runtimes (e.g., high-fidelity postoperative evaluation, retrospective simulation studies, or research workflows with sparse EMG). It is not intended for real-time control or closed-loop applications. For the Arm26 benchmark (T=528), we use MATLAB paretosearch with ParetoSetSize=30 and a fixed function-evaluation budget. We use parallel objective evaluations when available. Local refinement is performed with patternsearch after Pareto-point selection. We report budgets for reproducibility because runtime depends strongly on hardware, OpenSim settings, and parallelization. Full solver settings are available from the corresponding author upon reasonable request (within the scope necessary to reproduce the reported results). As a concrete reference, on a workstation (Intel i7-10700K CPU (Intel, Santa Clara, CA, USA); MATLAB R2024b), the Pareto stage with a 90,000-evaluation budget took 201 min wall-clock using parallel objective evaluations (8 workers). This corresponds to an average OpenSim cost of ≈(201×60×8)/90,000≈1.07 s per objective evaluation (per worker), or ≈2 ms per time sample per evaluation in this Arm26 setting. Scaling beyond Arm26 is limited by both decision dimension and forward-model cost. Optimizing unmeasured activations at each sample yields $n=M_{\mathrm{miss}}T$ decision variables. Wall-clock time grows roughly with $N_{\mathrm{eval}}\times c\left(M,T\right)$ (amortized across workers), where c(M,T) is the OpenSim forward cost per evaluation. If c(M,T) is approximately linear in both muscle count and time samples, per-evaluation cost scales roughly with M×T. A lower-limb gait model with O(80) muscles and O(2000) samples could therefore increase per-evaluation cost by ∼(80/6)×(2000/528)≈50×. Naive 90,000-evaluation derivative-free searches would then be impractical. A practical path to scaling is therefore to reduce both (i) the decision dimension and (ii) the required number of evaluations. Practical options include warm-starting from SynRc/SO, optimizing directly in a reduced basis (synergy coefficients or spline knots), and using multi-fidelity/surrogate strategies to limit expensive OpenSim calls. As a small illustration, the joint-moment-only smoothness baseline (Table 5) is solved in a 20-dimensional knot parameterization with a 3000-evaluation budget in ≈5.5 min on the same workstation, showing that reduced-dimension parameterizations can make time-coupled refinement problems substantially cheaper.

### 4.5. Minimal Extensibility Demo: Reduced-Dimension Parameterization

**Figure 11:**
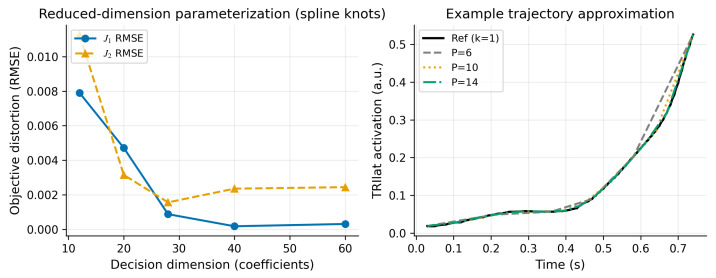
Compressing unmeasured activations distorts $\left(J_{1},J_{2}\right)$ unless enough coefficients are used. (**Left**): objective distortion versus number of knot coefficients *P* (evaluated on the fixed stage-I set); (**right**): example TRIlat trajectory approximation for one stage-I solution.

To illustrate that the framework is compatible with lower-dimensional decision variables (without switching to a larger musculoskeletal model), we construct a simple reduced-dimension parameterization for the unmeasured activations. Specifically, for each unmeasured muscle we represent $A^{\mathrm{miss}}\left(t\right)$ by a small number of knot values and reconstruct the full time series by interpolation, reducing the decision dimension from $M_{\mathrm{miss}}T$ to $M_{\mathrm{miss}}P$ coefficients. Using the fixed stage-I solutions as reference, we quantify how this compression distorts the objectives by re-evaluating $\left(J_{1},J_{2}\right)$ after compressing each candidate trajectory. Figure 11 shows that objective distortion decreases as the number of coefficients increases, demonstrating a concrete pathway to reduce decision dimension while preserving the trade-off geometry. This demo does not replace full re-optimization under a reduced parameterization. Instead, it provides a reproducible sanity check. It also motivates future work that solves the multi-objective problem directly in a lower-dimensional basis (i.e., re-running stage-I and/or stage-II over coefficients rather than over $M_{\mathrm{miss}}T$ samples). While OpenSim evaluation cost per function call remains, reducing the decision dimension can improve the efficiency of derivative-free search and makes neighborhood and refinement sensitivity analyses more tractable in larger models.

## 5. Conclusions

We presented a synergy-informed, joint-moment-consistent framework for reconstructing unmeasured muscle activations under sparse observations. The method combines (i) a SynRc prior derived from NMF synergies and bounded quadratic programming and (ii) a bound-constrained multi-objective optimization that enforces agreement with inverse-dynamics joint moments while retaining neural consistency. The stage-I Pareto set provides an interpretable trade-off between objectives, enabling diagnostic, task-dependent solution selection. For sparse-measurement studies without ground truth, the Pareto geometry and the measured-only SynRc diagnostic $E_{\mathrm{meas}}$ provide an interpretable view of identifiability and prior transferability. The recommended reporting protocol (Table 7), together with neighborhood and parameter sensitivity checks (Supplementary Table S4 and Supplementary Figure S3), offers a reproducible way to document selection choices and their stability. Accordingly, the main practical output is not a single “best” reconstruction, but a set of diagnostics that makes assumptions and trade-offs transparent. Synthetic studies demonstrate robustness to noise and mismatch, reveal how the trade-off geometry changes across observability regimes, and illustrate a reduced-dimension parameterization pathway for scaling. In the Arm26 benchmark, $J_{1}$-dominant selections reduced joint-moment error from 0.154 (SynRc-only) to ≈0.138, while more neural-consistent selections preserved the synergy prior and yielded near-ground-truth reconstruction for TRIlat (RMSE $\approx 10^{-3}$). These findings should be interpreted in light of the study limitations: verification is synthetic, evaluations assume fixed kinematics, and inverse-dynamics targets represent net moments that may include non-muscle contributions. Future work will focus on experimental sparse-EMG applications with subject-specific models and on scalable implementations using reduced-dimension bases and surrogate strategies.

## Figures and Tables

**Table 1:** Solver settings and hyperparameters used in this study (unless otherwise stated).

Item	Value	Description
Model	Arm26 (2 DOF, 6 muscles)	OpenSim upper-extremity benchmark (Section 2.9).
Time horizon	T=528 (0.71 s)	Activation grid used for all objectives.
Measured set	TRIlong, TRImed, BIClong, BRA	“Observed” channels fixed during reconstruction.
Unmeasured set	TRIlat, BICshort	Decision variables in the inverse problem.
Synergy rank	r=4	NMF synergy dimension.
SynRc QP weights	α=10, $\lambda_{\mathrm{syn}}=0.01$, $\gamma_{\mathrm{syn}}=0.1$	Measured-channel fit, Tikhonov regularization, and temporal excitation smoothing (Equation (4); distinct from the refinement weights λ and γ in Equation (13)).
ID preprocessing	4th-order Butterworth, 5 Hz, zero-phase	Low-pass filter applied to $\tau_{\mathrm{ID}}\left(t\right)$ (Section 2.9).
Stage-I solver	paretosearch	ParetoSetSize=30, MaxFunctionEvaluations=90,000, MeshTolerance=0.2.
Stage-II solver	patternsearch	MaxIterations=60, FunctionTolerance=$10^{-4}$; refinement objective in Equation (13).
Smoothness weights	γ∈{10,100,1000}	Representative sweep in Supplementary Figure S3.
Joint-moment-only baseline	P=10, γ=100, $N_{\mathrm{eval}}=3000$	Reduced-dimension knot parameterization used to solve $\min(J_{1}+\gamma J_{3})$ without a synergy prior (reported later in the Section 3).

**Table 2:** Case 2 (alternative measured set) yields nearly identical solutions across SynRc-only, stage-I selection, and refinement. Columns report joint-moment error $J_{1}$ (NRMSE) and unmeasured-muscle activation RMSE. “SO”: OpenSim static optimization (measured channels clamped for joint-moment evaluation); “SynRc-only”: SynRc prior without joint-moment-consistency optimization; “Stage-I”: selected stage-I point; “Opt”: refined solution.

Method	$J_{1}$ (NRMSE)	TRImed RMSE	BRA RMSE
SO	0.2914	0.07553	0.001286
SynRc-only	0.1414	0.0009898	0.00122
Stage-I ($k^{★}=2$)	0.1414	0.0009898	0.001277
Opt ($k^{★}=2$)	0.1414	0.0009898	0.001277

**Table 3:** Case 3 (synthetic gait benchmark) shows a non-degenerate trade-off and differing unmeasured-muscle RMSE across baselines and selections. Columns report joint-moment error $J_{1}$ (NRMSE) and unmeasured-muscle activation RMSE. Synthetic setup: $\tau_{\mathrm{ID}}$ is generated from the linearized moment map; unmeasured $F^{\max}$ is scaled by ×2. “SynRc-only”: SynRc prior without joint-moment-consistency optimization; “Joint-moment-only”: minimize $J_{1}+\gamma J_{3}$ without the synergy prior; “Stage-I”: selected stage-I point; “Opt”: refined solution.

Method	$J_{1}$ (NRMSE)	hamstrings_r RMSE	gastroc_r RMSE
SynRc-only	0.1454	0.03085	0.03126
Joint-moment-only	0.1463	0.03077	0.03126
Stage-I ($k^{★}=4$)	0.1432	0.03029	0.03126
Opt ($k^{★}=4$)	0.1465	0.03089	0.03126

**Table 4:** Observability regimes change the $J_{1}$ spread within the stage-I candidate set and the resulting selection $k^{★}$. Using the fixed baseline stage-I decision set, we report the selected index $k^{★}$, the empirical $J_{1}$ range under the modified forward model, and unmeasured-muscle RMSE at the selected point.

Scenario	Scale	$k^{★}$	$J_{1}$ range	$J_{1}\left(k^{★}\right)$	TRIlat RMSE	BICshort RMSE
Unmeasured strength scale ×0.2	0.2	22	[0.4250, 0.4251]	0.4251	0.0009	0.0332
Unmeasured strength scale ×1.0	1.0	16	[0.1532, 0.1539]	0.1535	0.0009	0.0335
Unmeasured strength scale ×2.0	2.0	30	[0.3319, 0.3337]	0.3327	0.0009	0.0329

**Table 5:** Objective values summarize the mechanics–prior trade-off across baselines and selected solutions. “CMC”: synthetic reference from computed muscle control; “SynRc-only”: SynRc prior without joint-moment-consistency optimization (so $J_{2}=0$ by definition); “Joint-moment-only”: minimize $J_{1}+\gamma J_{3}$ in a reduced-dimension parameterization (with $J_{2}$ reported post hoc); “Opt”: proposed method after local refinement.

Method/Setting	$J_{1}$ (NRMSE)	$J_{2}$ (RMSE)	$J_{3}$ (a.u.)
CMC reference	0.1414	0.0233	0.00205
SynRc-only baseline	0.1542	0	0.00316
Joint-moment-only + smoothness (P = 10 knots)	0.1449	0.0211	0.000918
Opt (stage-I point 1, γ=10)	0.1380	0.0280	0.0116
Opt (stage-I point 1, γ=100)	0.1382	0.0187	0.00607
Opt (stage-I point 18, γ=100)	0.1537	0.00174	0.00204
Opt (stage-I point 1, γ=1000)	0.1398	0.0266	0.00720

**Table 6:** Unmeasured-muscle RMSE varies across baselines and selected solutions. RMSE is computed versus synthetic ground truth; “Opt” denotes the proposed multi-objective method after local refinement. Bold marks the best (minimum) RMSE per column; underline marks the second-best when unique.

Method/Setting	TRIlat RMSE	BICshort RMSE
Static optimization (SO)	0.0754	0.0418
SynRc-only prior	**0.0009**	0.0329
Joint-moment-only + smoothness (P = 10 knots)	0.0284	**0.0324**
Opt (stage-I point 1, γ=10)	0.0395	0.0329
Opt (stage-I point 1, γ=100)	0.0262	0.0329
Opt (stage-I point 18, γ=100)	0.0010	0.0329
Opt (stage-I point 1, γ=1000)	0.0374	0.0329

**Table 7:** Protocol report template for applications without ground truth. Use this fill-in summary to report $E_{\mathrm{meas}}$, ID-quality diagnostics, the stage-I selection, and neighborhood sensitivity. Heuristic “flags” are grounded in the synthetic benchmarks (Supplementary Tables S1–S4) and should be interpreted as relative warnings rather than universal thresholds.

Item	Report (with Heuristic Flags)
$E_{\mathrm{meas}}$ (SynRc diagnostic)	Mean measured-channel RMSE Equation (5); interpret *relatively* across proxy-library choices. *Heuristic:* a large increase (e.g., >3× the lowest $E_{\mathrm{meas}}$ among candidate priors) often indicates proxy mismatch, motivating a shift toward lower $J_{1}$.
ID quality	Report HF ratio, spike score, and estimated lag (Supplementary Table S3), plus $J_{1,\min}$ (minimum achievable $J_{1}$ within the stage-I set). *Heuristic:* HF ratio ≳0.2, spike score ≳10, or |lag|≳5 ms are warning signs of potential ID artifacts, motivating a shift toward lower $J_{2}$.
Selection	Report the selection rule (endpoint, knee/utopia-closest) and the selected index $k^{★}$ with $\left(J_{1}\left(k^{★}\right),J_{2}\left(k^{★}\right)\right)$; also report the endpoints $\min J_{1}$ and $\min J_{2}$ on the stage-I set for context.
Δk sensitivity	Report a small neighborhood (e.g., $k^{★}\pm 2$) with $\left(J_{1},J_{2},J_{3}\right)$ (Supplementary Table S4); if stage-II refinement is used, also report a small γ sweep (Supplementary Figure S3) to show whether refinement conclusions are stable.

## Data Availability

The OpenSim musculoskeletal model files used in this study are publicly available from the OpenSim official website. The scripts, derived numerical results, and processed benchmark outputs used to generate the figures and tables are available from the corresponding author upon reasonable request (within the scope necessary for academic use and reproduction of the reported results).

## Supplementary Materials

The following supporting information can be downloaded at https://www.mdpi.com/article/10.3390/bioengineering13030293/s1, Figure S1: Synthetic robustness stress-tests under noise and mismatch; Figure S2: Reconstructed unmeasured activations across representative optimized configurations; Figure S3: Refinement sensitivity to the smoothness weight γ; Table S1: Robustness summary at the selected point; Table S2: Robustness statistics across random seeds; Table S3: ID-quality metrics for synthetic perturbations; Table S4: Neighborhood sensitivity around the utopia-closest selection.
