# Supplementary material for: Pareto-Based Diagnostics and Selection for Mechanics–Synergy Trade-Offs in Unmeasured Muscle Activation Reconstruction
Source: Bioengineering (Basel). 2026 Mar 1;13(3):293. doi: 10.3390/bioengineering13030293 (PMC13023886; doi:10.3390/bioengineering13030293)
Supplement: Supplementary file 1 [file bioengineering-13-00293-s001.zip › bioengineering-4125481-supplementary.pdf]

Supplementary Materials: Pareto-Based Diagnostics and Selection for Mechanics–Synergy Trade-Offs in Unmeasured Muscle Activation Reconstruction

Supplementary Figures

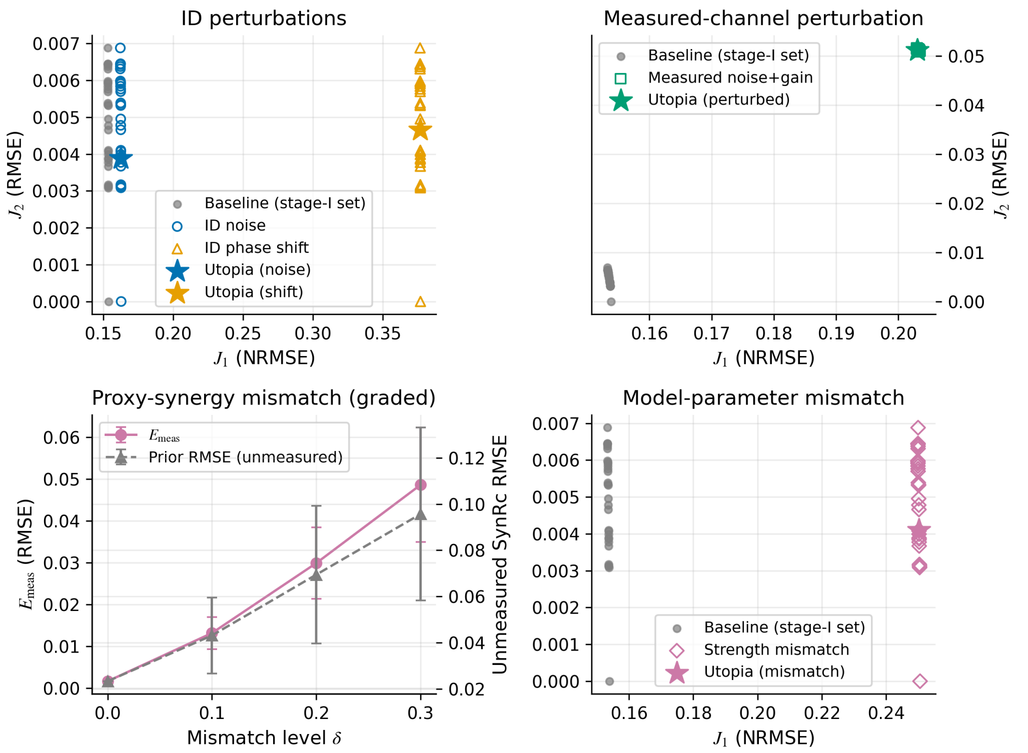

Figure S1. Robustness diagnostic: noise and mismatch can shift Pareto-point selection and inflate E_{meas}. Panels re-evaluate (J_1, J_2) on a fixed stage-I candidate set under (top-left) ID perturbations, (top-right) measured-channel noise+gain, (bottom-right) forward-model strength mismatch, and (bottom-left) graded proxy-synergy mismatch (showing E_{meas} and prior RMSE). Stars indicate utopia-closest selections.

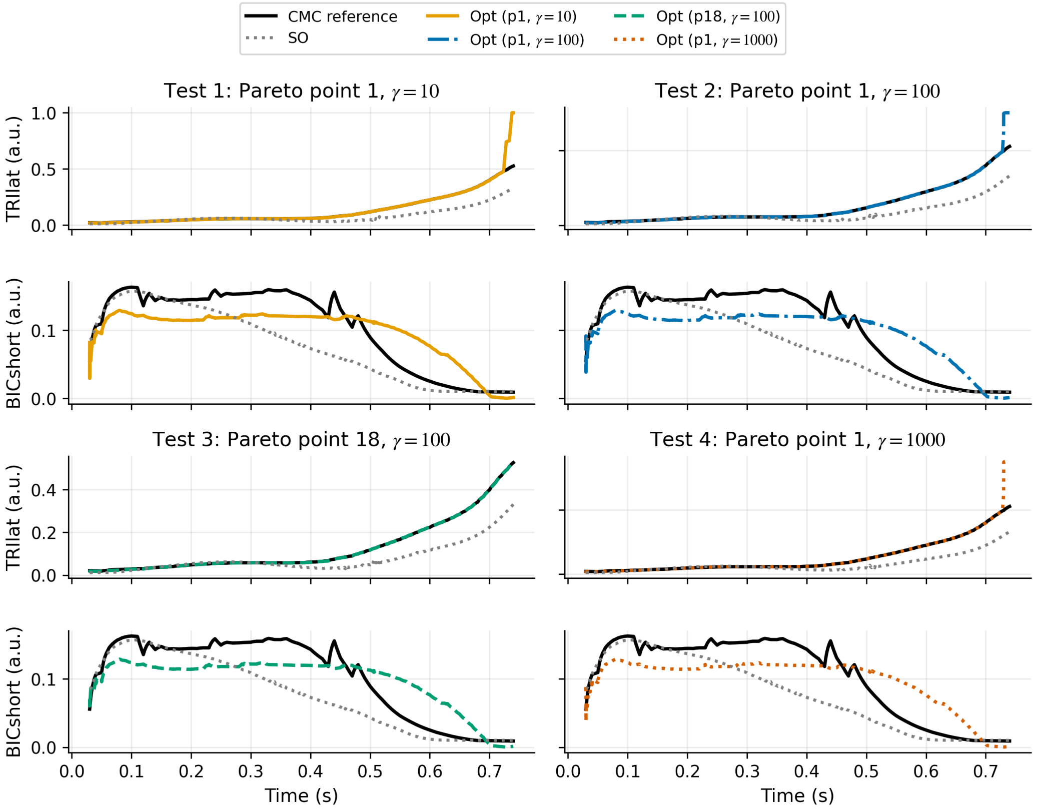

Figure S2. Optimized reconstructions vary with stage-I selection and γ. Panels show Opt versus SO and ground truth for Test 1 (point 1, $\gamma = 10$), Test 2 (point 1, $\gamma = 100$), Test 3 (point 18, $\gamma = 100$), and Test 4 (point 1, $\gamma = 1000$).

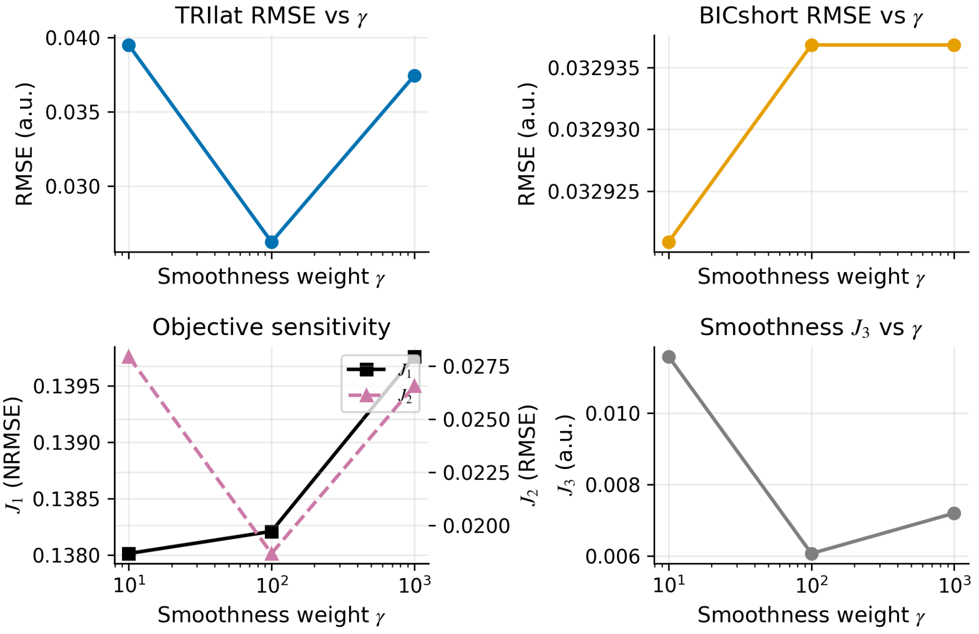

Figure S3. Refinement sensitivity: γ changes the balance among J_1, J_2, and smoothness J_3. Points show a discrete sweep $\gamma \in \{10, 100, 1000\}$ at stage-I point 1 (lines connect points for visual guidance).

Supplementary Tables

Table S1. Robustness perturbations can shift the selected index $k^\star$ and inflate diagnostics and RMSE. Using the fixed baseline stage-I decision set, we re-evaluate objectives and report $k^\star$ (utopia-closest rule), measured-channel SynRc error E_{meas}, and unmeasured-muscle RMSE versus synthetic ground truth.

Scenario	E_{meas}	$k^\star$	J_1	J_2	TRIlat RMSE	BICshort RMSE
Baseline	0.00162	16	0.1535	0.0050	0.0009	0.0335
ID noise (5% RMS)	0.00162	22	0.1626	0.0039	0.0009	0.0332
ID phase shift (5 ms)	0.00162	18	0.3770	0.0047	0.0009	0.0337
Measured noise+gain	0.00884	2	0.2031	0.0512	0.0009	0.0342
Model strength mismatch (±10%)	0.00162	19	0.2502	0.0041	0.0009	0.0332
Synergy mismatch ($\delta = 0.10$)	0.00809	18	0.1536	0.0557	0.0009	0.0337
Synergy mismatch ($\delta = 0.30$)	0.028	1	0.1532	0.1155	0.0009	0.0348

Table S2. Multi-seed robustness diagnostics quantify selection variability under stochastic perturbations. Using the fixed baseline stage-I decision set, we report mean±SD over random seeds for E_{meas}, objectives, and unmeasured-muscle RMSE at the selected point, and summarize the selected index $k^\star$ as mode (min–max).

Scenario	N	$k^\star$	E_{meas}	J_1	J_2	BICshort RMSE
ID noise (5% RMS)	20	21 (16–25)	0.00162	0.1622±0.0014	0.0040±0.0003	0.0332±0.0001
Measured noise+gain	5	10 (10–30)	0.00834±0.00055	0.2603±0.0377	0.0509±0.0034	0.0334±0.0003
Model strength mismatch (±10%)	5	16 (16–29)	0.00162	0.1716±0.0438	0.0041±0.0007	0.0334±0.0002
Synergy mismatch ($\delta = 0.10$)	20	7 (7–19)	0.0131±0.0039	0.1534±0.0001	0.0389±0.0169	0.0336±0.0001
Synergy mismatch ($\delta = 0.30$)	20	18 (1–19)	0.0486±0.014	0.1535±0.0001	0.0912±0.0404	0.0339±0.0005

Table S3. ID-quality metrics summarize the synthetic ID perturbations used in Figure S1. HF ratio: fraction of signal power above 10 Hz; spike score: $\max|\Delta\tau|/\text{RMS}(\Delta\tau)$; lag: time shift that best aligns the perturbed and baseline (z-scored) elbow moments; $J_{1,\min}$: minimum achievable joint-moment error within the fixed stage-I candidate set.

Scenario	HF ratio	Spike score	Lag (ms)	$J_{1,\min}$
Baseline	0.045	2.83	0	0.1532
ID noise (5% RMS)	0.102	3.3	0	0.1623
ID phase shift (5 ms)	0.579	18	-5	0.3768

Table S4. Neighboring stage-I Pareto points can change smoothness and RMSE even when J_1 varies little. Around the utopia-closest selection rule, we report (J_1, J_2) on the stage-I set, smoothness J_3 (from unmeasured activations), and unmeasured-muscle RMSE versus synthetic ground truth. Indices k are sorted by decreasing J_2 (increasing neural consistency), so $k^\star \pm 2$ denotes adjacent solutions in that ordering.

Δk	k	J_1	J_2	J_3	TRIlat RMSE	BICshort RMSE
−2	14	0.1535	0.005339	0.007914	0.0009409	0.03366
−1	15	0.1535	0.005319	0.007882	0.0009409	0.03366
+0	16	0.1535	0.004954	0.006258	0.0009409	0.0335
+1	17	0.1536	0.00478	0.00618	0.0009409	0.03345
+2	18	0.1536	0.00466	0.007	0.0009409	0.03371
